# Gait-Based Screening for Cognitive Impairment in Older Adults: A Fast and Objective Approach

**DOI:** 10.3390/healthcare13192450

**Published:** 2025-09-26

**Authors:** Jose Luis Perez-Lasierra, Marina Azpíroz-Puente, Martin Morita-Hernandez, Antonio Gómez-Bernal, José-Víctor Alfaro-Santafé, Javier Alfaro-Santafé

**Affiliations:** 1 Podoactiva Biomechanical Unit, Research & Development Department, Parque Tecnológico Walqa, Ctra. N330a Km 566, 22197 Cuarte, Spain; marinaazpiroz@podoactiva.com (M.A.-P.); martinmorita@podoactiva.com (M.M.-H.); antoniogomez@podoactiva.com (A.G.-B.); victoralfaro@podoactiva.com (J.-V.A.-S.); javieralfaro@podoactiva.com (J.A.-S.); 2 Facultad de Ciencias de la Salud, Universidad San Jorge, Villanueva de Gállego, 50830 Zaragoza, Spain; 3 Department of Podiatry, Faculty of Health Sciences, Manresa University, 08243 Manresa, Spain

**Keywords:** cognitive decline, gait analysis, spatiotemporal parameters, early screening, elderly

## Abstract

Background/Objectives: Cognitive impairment in older adults is a growing public health concern due to global population aging. Early detection is crucial, yet current screening methods are time-consuming and require clinical expertise. Gait analysis has emerged as a promising alternative for cognitive screening. The aim of the study was to identify gait variables associated with cognitive impairment and to develop predictive algorithms capable of distinguishing between cognitively impaired and non-impaired older adults using gold-standard gait analysis. Methods: A cross-sectional study was conducted with 42 adults aged > 60 years. Cognitive function was assessed using the Mini-Mental State Examination (MMSE), and participants were divided into high (MMSE > 24) and low (MMSE ≤ 24) cognitive function groups. Spatiotemporal gait parameters were recorded at participants’ usual pace using the Optogait system. Logistic regression models were developed using half of the sample (training group) and validated in the remaining participants (verification group). Results: Algorithms based on stride length and double support time demonstrated high classification performance. In the training group, the best-performing model achieved an AUC-ROC of 0.91, with a sensitivity of 71.4% and specificity of 92.3%. Validation in the verification group yielded an AUC-ROC of 0.84 and accuracy of 81.0%. Alternative models showed acceptable to excellent predictive power. Conclusions: Gait analysis using gold-standard methods can effectively identify cognitive impairment in older adults. The developed algorithms offer a rapid, objective, and accurate screening alternative with strong potential for clinical application.

## 1. Introduction

The global elderly population is increasing, representing a new demographic reality. While population aging was first observed in developed countries several decades ago, this phenomenon is now also affecting developing nations [1]. Current projections estimate that by 2050, approximately 2.1 billion people will be aged 60 years or older, accounting for 21% of the world’s population [2].

As people age, both brain structure and cognitive function undergo deterioration, increasing the likelihood of developing various pathologies [3,4]. Among the conditions most commonly associated with cognitive impairment in older adults are mild cognitive impairment (MCI) in the early stages, and Alzheimer’s disease (AD) and other forms of dementia in more advanced stages of cognitive decline [5]. Given the global aging trend, the incidence and prevalence of these conditions are expected to rise in the coming years [6], underscoring the critical importance of early prevention and the development of effective screening tools.

Currently, screening for these types of diseases is conducted during medical consultations using standardized questionnaires [7,8,9]. However, considering the projected increase in the number of cases in the coming years, along with the existing shortage of healthcare personnel to meet the rising demand on healthcare systems [10], the current screening approach is neither sustainable nor efficient [7]. As a result, many cases are not diagnosed until the disease has progressed to more advanced stages [8].

In response to this issue, alternative screening approaches have emerged in recent years [11,12,13], some of which are based on gait analysis [14]. Most studies employ inertial measurement units (IMUs) [15,16,17], which offer advantages such as portability and potential applicability in real-world settings [18]. However, the use of inertial systems for screening cognitive diseases through gait analysis still requires further investigation, as concerns remain regarding their reliability and validity [19]. Despite their potential for future deployment in real-world environments, the fully autonomous use of these systems currently poses challenges and may lead to inaccurate conclusions, as they often depend on data that may contain errors or noise.

However, the accurate assessment of gait and its parameters remains highly relevant in older adults, and its implementation has become a pressing necessity. Numerous studies have demonstrated that gait-related variables are important indicators of health status and functional capacity, with some even being considered the “sixth vital sign” [20,21,22,23]. Moreover, gait has been shown to be sensitive to neurological and cognitive interventions [24,25]. In the short term, an objective and precise gait assessment system could provide substantial value and play a key role in clinical evaluations. Therefore, establishing associations between gait parameters—measured using reference standard systems—and the presence of highly prevalent, age-related conditions such as cognitive decline is of particular interest.

We hypothesize that specific gait parameters are significantly associated with the presence of cognitive impairment and that these parameters can be used to develop reliable algorithms capable of distinguishing between individuals with and without cognitive impairment. Therefore, the aim of this study was to investigate which gait parameters are associated with cognitive impairment and to develop algorithms capable of distinguishing between individuals with and without cognitive impairment based on gait parameters assessed by gold-standard methods.

## 2. Materials and Methods

### 2.1. Study Design and Recruitment

A cross-sectional study was conducted using a convenience sampling approach. Individuals over 60 years of age with a diagnosis of cognitive impairment were recruited from the Rey Ardid Foundation in Zaragoza, Spain. Control participants (without a prior diagnosis of cognitive impairment), also over 60 years of age, were recruited from adult daycare centers and among community-dwelling, able-bodied individuals living in Zaragoza through word-of-mouth. To be eligible for the study, participants were required to be able to walk independently without assistance and to have no medical conditions or disorders that could affect gait, including other neurological diseases or mobility disorders. Individuals with any type of prosthesis in the lower limbs were also excluded from participation.

All procedures were carried out in accordance with relevant guidelines and regulations. The study was approved by the Clinical Research Ethics Committee of Aragon (CEICA) (PI23/084), and written informed consent was obtained from all participants.

### 2.2. Cognitive Function Assessment

Cognitive function was assessed using the Spanish version of the Mini-Mental State Examination (MMSE) [26]. The MMSE consists of 11 items and is widely used in both clinical and research settings to evaluate cognitive performance across various domains. The total score ranges from 0 to 30, with higher scores indicating better cognitive function. Based on previously published literature, an MMSE score of ≤24 points was considered indicative of low cognitive function [26].

Participants were classified into two groups: a high cognitive function (HCF) group (MMSE > 24), and a low cognitive function (LCF) group, defined by either an MMSE ≤ 24 or the presence of a clinical diagnosis of cognitive impairment. From the total sample, 50% of the participants in both the HCF and LCF groups were randomly selected to form Group 1, which was used to develop the detection algorithm (training dataset). The remaining participants constituted Group 2, which was used to evaluate the sensitivity and specificity of the algorithms developed using Group 1.

### 2.3. Gait Analysis

**Figure 1:**
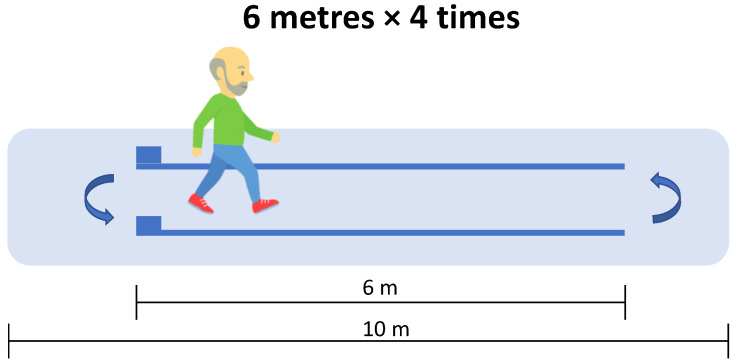
Gait analysis performed by participants walking straight using the Optogait system.

The Optogait system (Microgate, Bolzano, Italy) was used to assess spatiotemporal gait variables. This system consists of two parallel modular-length bars equipped with photocells that detect foot contact events at a sampling frequency of 1000 Hz. The Optogait system has been previously validated for assessing spatiotemporal gait parameters in various populations, including older adults [27]. The variables assessed included gait speed, cadence, stride length, stance time, swing time, single support time, double support time, contact phase, foot flat, propulsive phase, and gait ratio. Detailed definitions and explanations of these variables are provided in the supplementary material (Table S1). Participants were instructed to start from a standing position and walk at a normal, self-selected pace along a 6 m path delineated by the parallel Optogait bars, which were positioned in the middle of a 10 m corridor (Figure 1). Each participant completed four traverses of the corridor, performing turns at both the starting and ending points.

### 2.4. Statistical Analysis

Continuous variables are presented as the mean and standard deviation (SD), while categorical variables are expressed as frequencies and percentages. All variables were complete, and no missing data were present in the dataset. Independent samples *t*-tests and Chi-square tests were used to examine baseline differences between Group 1 (training dataset) and Group 2 (verification dataset). Gait variables were entered into multivariate logistic regression models, with low cognitive function as the dependent variable. Forward conditional stepwise selection was employed in Model A. Additional regression models (Models B and C) using the forced entry method were also evaluated, with independent variables selected based on differences between the LCF and HCF groups. Relevant diagnostic analyses were conducted to ensure that no collinearity existed among the gait parameters included in each model. The discriminatory ability of the models was assessed using the area under the receiver operating characteristic curve (AUC-ROC). Model calibration was evaluated using the Hosmer-Lemeshow test (Table S3). The optimal cut-off point was determined by the maximal Youden Index to evaluate model accuracy. AUC values were interpreted as follows: 0.5 indicated no discrimination, 0.7–0.8 acceptable, 0.8–0.9 excellent, and >0.9 outstanding [28]. Statistical analyses were performed using SPSS statistical software version 29.0 (IBM Corp., Armonk, NY, USA) and R statistical software (version 4.3.1.).

## 3. Results

### 3.1. Descriptive Data

Descriptive data for the participants, stratified by cognitive function and for the overall sample, are presented in Table 1. Additionally, participant characteristics according to group allocation (Group 1: training dataset; Group 2: verification dataset) are shown in Table 2. Statistically significant differences between the LCF and HCF groups were found in mean age, gait speed, stride length, stance phase, contact phase, and propulsive phase (Table 1). In contrast, no significant differences were observed in baseline characteristics between Group 1 and Group 2 (Table 2), indicating adequate randomization of the sample.

### 3.2. Algorithm Development Through Logistic Regression Models

**Figure 2:**
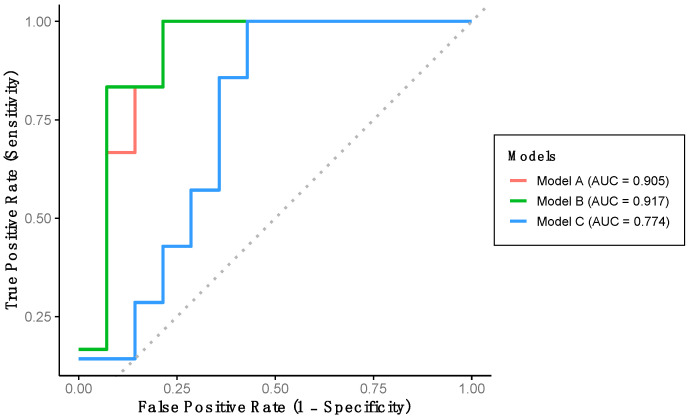
Performance of the developed models using training dataset. Model A includes stride length and percentage of double support time. Model B includes stride length, percentage of double support time and percentage of stance phase. Model C includes stride length, gait speed and percentage of stance phase.

Using the training dataset (Group 1), a multivariate logistic regression model with backward stepwise selection was conducted to develop an algorithm capable of classifying participants according to cognitive function level. Among all gait variables analyzed, the final algorithm (Model A) includes stride length and percentage of double support time as significant predictors. Model A achieved a sensitivity of 71.4%, a specificity of 92.3%, and an overall predictive accuracy of 85.0% (Table 3). This model demonstrates an outstanding discriminatory performance for detecting LCF, as evidenced by the AUC-ROC (Figure 2).

Indeed, a multivariate logistic regression model with a forced entry method was conducted. We first constructed Model B, which includes the variables in Model A and additionally the percentage of stance phase variable (Table 3). This model achieved a sensitivity and specificity of 71.4% and 92.3%, respectively, with a predictive accuracy of 85.0% (Table 3). Model B also demonstrated outstanding discriminative performance for detecting LCF, as indicated by the AUC-ROC (Figure 2). Additionally, Model C was developed, incorporating stride length, gait speed, and percentage of stance phase. This model yielded a sensitivity of 40.0%, a specificity of 73.3%, and a predictive accuracy of 65.0% (Table 3). This model shows an acceptable AUC-ROC for LCF detection (Figure 2).

### 3.3. Validation of Classification Models Using an Independent Dataset

**Figure 3:**
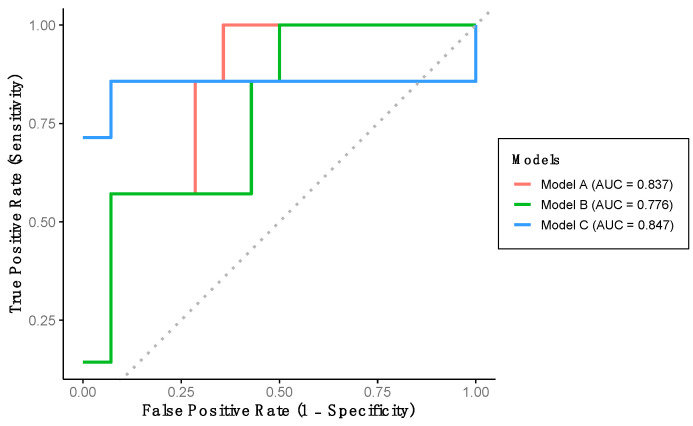
Performance of the developed models using verification dataset. Model A includes stride length and percentage of double support time. Model B includes stride length, percentage of double support time and percentage of stance phase. Model C includes stride length, gait speed and percentage of stance phase.

The performance of the previously developed classification models was evaluated using the verification dataset (Group 2). Model A achieved a sensitivity of 57.1%, a specificity of 92.9%, and a predictive accuracy of 81.0% (Table 3). This model demonstrated excellent discriminative ability for detecting LCF, as indicated by the AUC-ROC (Table 3, Figure 3). Model B yielded identical sensitivity, specificity, and accuracy values as Model A; however, the AUC-ROC was categorized as acceptable rather than excellent (Table 3, Figure 3). Finally, Model C achieved a sensitivity of 85.7%, a specificity of 57.1%, and a predictive accuracy of 66.7%. Despite its lower specificity, this model also demonstrated excellent AUC-ROC performance for LCF detection (Table 3, Figure 3).

## 4. Discussion

The results of this study suggest that several gait parameters, assessed using gold-standard methods, are significantly associated with cognitive decline and may contribute to the development of classification algorithms capable of distinguishing individuals with and without cognitive impairment.

The findings of this study are consistent with previous literature, which indicates, among other aspects, that gait speed is lower in individuals with cognitive impairment compared to those without cognitive impairment [29]. Several studies have supported the notion that gait speed is one of the most relevant gait indicators and may serve as an early marker for cognitive decline [21,22]. However, based on the available scientific literature, the performance of gait speed alone as a screening tool for cognitive impairment is limited, with reported AUC-ROC values ranging between 0.51 and 0.74 [30,31]. While our results align with previous studies and underscore the importance of gait speed as a key variable for screening, the broader findings of our study suggest that combining additional, often-overlooked gait parameters through algorithmic approaches, could provide more accurate indicators or predictors of conditions such as cognitive impairment. The developed algorithms indicate that variables such as stride length and percentage of double support time may serve as effective indicators of cognitive dysfunction, a conclusion supported by other studies as well [32,33,34]. Typically, an increased double support time is associated with a compensatory strategy aimed at enhancing stability in response to gait instability [32], whereas a reduced stride length reflects diminished range of motion and a stiffer gait pattern characterized by limited joint mobility.

The developed algorithms showed promising performance in detecting cognitive impairment compared to many of the screening tests currently used in routine clinical practice [35,36,37]. For example, the Montreal Cognitive Assessment (MoCA), which is widely used and globally recognized for screening individuals with cognitive impairment, achieves an AUC of 0.80 [35]. Other commonly used screening tools in primary care settings, such as the Clock Drawing Test (CDT) and the Informant Questionnaire (IQ), yield AUC values of 0.83, and 0.77, respectively [36]. In addition to offering enhanced performance for detecting cognitive impairment, it is noteworthy that the gait test required to apply our algorithm can be completed in approximately one minute, representing a time reduction of over 85% compared with conventional cognitive assessments [37,38]. This aspect is particularly relevant in the context of the current overload faced by many healthcare systems worldwide [39]. Every minute saved translates into a substantial gain in efficiency, especially considering that the average duration of a primary care consultation in Europe is 10.7 min [40]. Moreover, beyond improved efficiency, the algorithms also offer greater efficacy, as their performance in detecting cognitive impairment surpasses that of traditional screening methods employed in clinical practice.

It is true that the economic cost of implementing gait analysis in clinical settings using gold-standard methods, such as the one employed in this study, would be higher than that associated with commonly used questionnaires or tests such as the MoCA or CDT. However, gait is a complex task that requires the integration and coordination of multiple physiological systems [41], and subtle alterations in gait patterns have been shown to be associated with abnormal or pathological functioning of one or more of these systems [41,42]. Gait analysis provides valuable information that could not only facilitate the screening of cognitive impairment but also support the early detection of a wide range of age-related diseases [43,44,45], whose screening currently demands considerable consultation time and thus incurs significant economic costs [8,38]. Implementing gait analysis in primary care could potentially replace several separate screening tests, thereby enhancing both efficiency and effectiveness, and might contribute to earlier diagnosis. This approach could not only support individual health outcomes but also offer potential economic advantages for healthcare systems by allowing the screening of multiple conditions through a single test. Early diagnosis would, in turn, enable the timely initiation of treatments when necessary, potentially avoiding more costly and prolonged interventions often required at advanced stages of disease. Therefore, while preliminary, our results suggest that while the initial cost of introducing gait analysis into primary care may seem high, the long-term benefits—both in terms of patient care quality and cost-effectiveness—could outweigh the initial investment by significantly reducing the time and resources required for screening. Future research should confirm these findings and further evaluate the feasibility of implementing gait analysis in real-world clinical practice.

In this study the predictive models were developed using gait parameters only, without including age or other demographic variables. This decision was intentional to generate tools capable of classifying cognitive impairment based solely on objective gait features. Although there was a statistically significant age difference between participants with and without cognitive impairment, all participants were older adults (≥60 years), and the age range remained relatively narrow. Therefore, the predictive value of gait parameters is evaluated within this older adult population. In addition, model validation was performed using an internal training/validation split. Future studies could explore integrating demographic variables such as age to potentially improve classification performance or disentangle age-related effects from cognitive.

This study presents notable strengths. The use of a gold-standard gait analysis system ensured high measurement precision and reliability. Additionally, the development and validation of the classification algorithms in independent subsamples enhance the robustness and potential clinical applicability of the findings. However, certain limitations should be acknowledged. First, the cross-sectional design does not allow for establishing causal relationships between gait alterations and cognitive impairment. Therefore, while associations can be observed, longitudinal studies are needed to determine causality and the temporal sequence of these changes. Second, the overall sample size was relatively small, and convenience-based recruitment may introduce selection bias, which may limit the generalizability of the findings. Future studies should aim to replicate these results in larger and more diverse cohorts to strengthen external validity and ensure broader applicability. However, the number of participants with cognitive impairment was sufficient to develop and validate robust classification algorithms. Third, baseline comparisons between groups were not adjusted for multiple testing, as these analyses were intended for descriptive purposes only. Therefore, the results of these comparisons should be interpreted with caution. Fourth, the models do not include age or other demographic information; therefore, some of the observed associations between gait parameters and cognitive impairment may reflect age-related differences rather than cognitive decline.

## 5. Conclusions

This study suggests that specific spatiotemporal gait parameters, objectively assessed using gold-standard methods, are significantly associated with cognitive impairment in older adults. Algorithms developed with these variables showed promising classification accuracy and outperformed traditional cognitive screening tools in both performance and time efficiency. These findings highlight the potential of gait analysis as a feasible, efficient, and effective approach for screening cognitive impairment in clinical settings, although further research is needed to confirm these results.

## Figures and Tables

**Table 1:** Baseline characteristics of study participants according to cognitive function.

	Overall	LCF	HCF	Effect Size (95% CI)	*p*-Value
**N**	42	14	28		
**Age**, years	72.38 (8.33)	78.07 (8.25)	69.54 (6.88)	−8.54 (−13.40, −3.67)	**0.001**
**Males**, %	38.1 [16]	42.9 [6]	35.7 [10]	0.74 (0.20, 2.75)	0.653
**Height**, cm	160.21 (8.54)	156.75 (9.53)	161.94 (7.60)	5.19 (−0.29, 10.66)	0.063
**Weight**, kg	68.95 (12.12)	64.79 (12.20)	71.04 (11.75)	6.25 (−1.86, 14.37)	0.116
**MMSE**, points	25.31 (5.82)	18.50 (5.23)	28.71 (1.49)	1.42 (7.16, 13.27)	**<0.001**
**Gait speed**, m/s	1.04 (0.26)	0.87 (0.25)	1.13 (0.23)	0.26 (0.11, 0.41)	**0.002**
**Cadence**, steps/min	107.77 (17.83)	105.92 (28.05)	108.69 (10.05)	2.77 (−13.75, 19.29)	0.641
**Stride length**, cm	117.61 (21.14)	102.86 (18.01)	124.98 (18.79)	22.11 (9.84, 34.38)	**<0.001**
**Stance time**, s	0.830 (0.300)	0.968 (0.472)	0.762 (0.122)	−0.21 (−0.39, −0.02)	**0.034**
**Stance time**, %	67.30 (6.40)	69.99 (9.64)	65.95 (3.44)	−4.04 (−8.12, 0.05)	0.053
**Swing time**, s	0.369 (0.082)	0.343 (0.127)	0.381 (0.045)	0.38 (−0.36, 0.11)	0.158
**Swing time**, %	32.70 (6.41)	30.02 (9.65)	34.04 (3.47)	4.02 (−0.76, 8.12)	0.054
**Single support**, s	0.368 (0.082)	0.343 (0.126)	0.381 (0.445)	0.39 (−0.04, 0.11)	0.149
**Single support**, %	33.66 (5.73)	31.42 (9.07)	34.69 (2.92)	3.27 (−0.52, 7.06)	0.089
**Double support**, s	0.346 (0.076)	0.360 (0.094)	0.338 (0.066)	−0.02 (−0.07, 0.03)	0.394
**Double support**, %	30.93 (4.86)	31.92 (4.82)	30.47 (4.90)	−1.45 (−4.76, 1.86)	0.382
**Contact phase**, s	0.076 (0.023)	0.069 (0.023)	0.080 (0.023)	0.01 (−0.01, 0.03)	0.161
**Contact phase**, %	9.92 (3.50)	8.25 (3.46)	10.76 (3.26)	2.51 (0.31, 4.71)	**0.027**
**Foot flat**, s	0.427 (0.107)	0.414 (0.147)	0.433 (0.083)	0.02 (−0.07, 0.11)	0.595
**Foot flat**, %	55.12 (13.91)	50.32 (20.14)	57.51 (8.97)	7.19 (−1.83, 16.22)	0.115
**Propulsive phase**, s	0.330 (0.357)	0.490 (0.562)	0.250 (0.147)	−0.24 (−0.47, −0.01)	**0.039**
**Propulsive phase**, %	34.98 (15.81)	41.45 (22.80)	31.75 (9.84)	−9.70 (−19.83, 0.43)	0.060
**Gait ratio**	2.033 (0.434)	2.160 (0.532)	1.974 (0.377)	−0.19 (−0.48, 0.11)	0.205

LCF: Low Cognitive Function; HCF: High Cognitive Function; MMSE: Mini-Mental State Examination. Values are mean (SD) or % [number]; Mean difference (95% CI) is reported for continuous variables, and relative risk (95% CI) is reported for categorical variables.

**Table 2:** Baseline characteristics of study participants according to group.

	Overall	TD	VD	Effect Size (95% CI)	*p*-Value
**N**	42	21	21		
**Age**, years	72.38 (8.33)	70.29 (6.92)	74.48 (9.22)	4.19 (−0.89, 9.27)	0.104
**Males**, %	38.1 [16]	47.6 [10]	28.6 [6]	0.44 (0.12, 1.58)	0.204
**Height**, cm	160.21 (8.54)	160.5 (7.57)	159.92 (9.60)	−0.57 (−5.97, 4.82)	0.831
**Weight**, kg	68.95 (12.12)	70.40 (10.38)	67.50 (13.75)	−2.90 (−10.50, 4.70)	0.446
**MMSE**, points	25.31 (5.82)	26.05 (4.97)	24.57 (6.61)	−1.48 (−5.12, 2.17)	0.418
**Gait speed**, m/s	1.04 (0.26)	1.07 (0.27)	1.02 (0.26)	−0.05 (−0.22, 0.11)	0.523
**Cadence**, steps/min	107.77 (17.83)	106.57 (11.53)	108.96 (22.71)	2.38 (−8.85, 13.62)	0.670
**Stride length**, cm	117.61 (21.14)	119.39 (20.20)	115.83 (22.39)	−3.56 (−16.86, 9.74)	0.592
**Stance time**, s	0.830 (0.300)	0.778 (0.118)	0.883 (0.406)	0.10 (−0.08, 0.29)	0.264
**Stance time**, %	67.30 (6.40)	66.07 (2.98)	68.52 (8.48)	2.46 (−1.51, 6.42)	0.218
**Swing time**, s	0.369 (0.082)	0.388 (0.032)	0.349 (0.110)	−0.04 (−0.09, 0.01)	0.127
**Swing time**, %	32.70 (6.41)	33.93 (3.00)	31.48 (8.49)	−2.45 (−6.42, 1.52)	0.219
**Single support**, s	0.368 (0.082)	0.388 (0.031)	0.349 (0.109)	−0.04 (−0.09, 0.01)	0.116
**Single support**, %	33.66 (5.73)	34.54 (2.52)	32.73 (7.78)	−1.82 (−5.44, 1.80)	0.316
**Double support**, s	0.346 (0.076)	0.354 (0.088)	0.337 (0.063)	−0.02 (−0.06, 0.03)	0.485
**Double support**, %	30.93 (4.86)	30.67 (5.02)	31.20 (4.81)	0.52 (−2.58, 3.63)	0.735
**Contact phase**, s	0.076 (0.023)	0.082 (0.024)	0.070 (0.021)	−0.01 (−0.03, 0.01)	0.093
**Contact phase**, %	9.92 (3.50)	10.76 (3.28)	9.09 (3.59)	−1.67 (−3.81, 0.47)	0.123
**Foot flat**, s	0.427 (0.107)	0.444 (0.100)	0.410 (0.114)	−0.03 (−0.10, 0.03)	0.313
**Foot flat**, %	55.12 (13.91)	57.37 (9.53)	52.87 (17.17)	−4.50 (−13.16, 4.16)	0.300
**Propulsive phase**, s	0.330 (0.357)	0.252 (0.119)	0.408 (0.484)	0.16 (−0.06, 0.38)	0.160
**Propulsive phase**, %	34.98 (15.81)	31.89 (10.50)	38.08 (19.55)	6.20 (−3.59, 15.98)	0.208
**Gait ratio**	2.033 (0.434)	1.971 (0.285)	2.098 (0.550)	0.13 (−0.15, 0.40)	0.356

TD: Training data; VD: Verification data; MMSE: Mini-Mental State Examination. Values are mean (SD) or % [number]; Mean difference (95% CI) is reported for continuous variables, and relative risk (95% CI) is reported for categorical variables.

**Table 3:** Statistical performance metrics of models according to data group.

	AUC-ROC (95% CI)	*p*-Value	Cut-Off Value	Accuracy	Sensitivity	Specificity
**TRAINING DATA**						
Model A	90.5 (77.0, 1.0)	<0.001	0.2567	85.0%	71.4%	92.3%
Model B	91.7 (78.8, 1.0)	<0.001	0.2552	85.0%	71.4%	92.3%
Model C	77.4 (57.0, 97.8)	0.009	0.1925	65.0%	40.0%	73.3%
**VERIFICATION DATA**						
Model A	83.7 (66.4, 1.0)	<0.001	-	81.0%	57.1%	92.9%
Model B	77.6 (56.7, 98.4)	0.010	-	81.0%	57.1%	92.9%
Model C	84.7 (58.9, 1.0)	0.008	-	66.7%	85.7%	57.1%
**OVERALL DATA**						
Model A	86.3 (75.2, 97.4)	<0.001	-	80.5%	76.9%	82.1%
Model B	84.3 (72.1, 96.5)	<0.001	-	82.9%	76.9%	85.7%
Model C	81.0 (65.3, 96.8)	<0.001	-	70.7%	92.3%	60.7%

AUC-ROC: Area under the receiver operating characteristic curve. Model A includes stride length and percentage of double support time. Model B includes stride length, percentage of double support time and percentage of stance phase. Model C includes stride length, gait speed and percentage of stance phase.

## Data Availability

The data presented in this study are available on request from the corresponding author due to ethical reasons.

## Supplementary Materials

The following supporting information can be downloaded at: https://www.mdpi.com/article/10.3390/healthcare13192450/s1, Table S1: Spatiotemporal parameters measured by Optogait system; Table S2: Multivariate logistic regression models for the association between gait parameters and low cognitive function; Table S3: Hosmer-Lemeshow test for model calibration.

## Abbreviations

The following abbreviations are used in this manuscript:

Abbreviation	Meaning
AD	Alzheimer’s disease
AUC-ROC	Area under the receiver operating characteristic curve
CDT	Clock drawing test
HCF	High cognitive function
IQ	Informant questionnaire
IMUs	Inertial measurement units
LCF	Low cognitive function
MCI	Mild cognitive impairment
MMSE	Mini-Mental state examination
MoCA	Montreal cognitive assessment
SD	Standard deviation
