# Supplementary material for: Gait-Based Screening for Cognitive Impairment in Older Adults: A Fast and Objective Approach
Source: Healthcare (Basel). 2025 Sep 26;13(19):2450. doi: 10.3390/healthcare13192450 (PMC12523893; doi:10.3390/healthcare13192450)
Supplement: Supplementary file 1 [file healthcare-13-02450-s001.zip › healthcare-3864972-supplementary.pdf]

Supplementary Material

Table S1. Spatiotemporal gait variables measured by Optogait system:

**Gait Speed**	The average speed at which the person walks, calculated as distance divided by time
**Cadence**	The number of steps taken per minute
**Stride length**	The distance between two successive contacts of the same foot
**Stance time**	The duration from heel contact to toe-off of the same foot
**Percentage of stance time**	The stance time expressed as a percentage of the total gait cycle for the same foot
**Swing time**	The time interval from toe-off to the next heel contact of the same foot – when the foot is in the air
**Percentage of swing time**	The swing time expressed as a percentage of the gait cycle
**Single support time**	The time during which only one foot is in contact with the ground
**Percentage of single support time**	The same single support time, expressed as a percentage of the gait cycle
**Double support time**	The total time during gait cycle when both feet are simultaneously in contact with the ground
**Percentage of double support time**	The double support time expressed as a percentage of the total gait cycle
**Contact Phase**	Time from heel contact to full flat on the ground. It is part of the stance phase
**Percentage of contact phase**	The contact phase expressed as a percentage of the total stance time
**Foot Flat**	Time during which the entire plantar surface of the foot is in contact with the ground
**Percentage of foot flat**	The foot flat time as a percentage of the total stance time
**Propulsive Phase**	Time from heel rise to toe-off, when the body is being propelled forward
**Percentage of propulsive phase**	The propulsive phase expressed as a percentage of stance phase
**Gait Ratio**	A derived measure that expresses the relationship between stride length and cadence

Table S2. Multivariate logistic regression models for the association between gait parameters and low cognitive function.

	OR (95%CI)	P-value
Model A		
Stride length	0.82 (0.68, 0.99)	0.047
% Double support	0.49 (0.19, 1.21)	0.124
Model B		
Stride length	0.80 (0.64, 0.99)	0.046
% Double support	0.51 (0.20, 1.26)	0.143
% Stance phase	0.86 (0.60, 1.25)	0.433
Model C		
Stride length	0.96 (0.90, 1.03)	0.295
Gait speed	0.16 (0.01, 241.34)	0.623
% Stance phase	1.02 (0.86, 1.20)	0.855

Table S3. Hosmer-Lemeshow test for model calibration.

	Hosmer-Lemeshow p-value
TRAINING DATA	
Model A	0.907
Model B	0.664
Model C	0.509
VERIFICATION DATA	
Model A	0.006
Model B	0.004
Model C	0.027
OVERALL DATA	
Model A	0.161
Model B	0.441
Model C	0.493
